# Selenium Status Is Positively Associated with Bone Mineral Density in Healthy Aging European Men

**DOI:** 10.1371/journal.pone.0152748

**Published:** 2016-04-07

**Authors:** Carolien M. Beukhof, Marco Medici, Annewieke W. van den Beld, Birgit Hollenbach, Antonia Hoeg, W. Edward Visser, Wouter W. de Herder, Theo J. Visser, Lutz Schomburg, Robin P. Peeters

**Affiliations:** 1 Department of Internal Medicine, Rotterdam Thyroid Center, Erasmus Medical Center, Rotterdam, The Netherlands; 2 Institut für Experimentelle Endokrinologie, Charité-Universitätsmedizin Berlin, Berlin, Germany; Tel Aviv Sourasky Medical Center, ISRAEL

## Abstract

**Objective:**

It is still a matter of debate if subtle changes in selenium (Se) status affect thyroid function tests (TFTs) and bone mineral density (BMD). This is particularly relevant for the elderly, whose nutritional status is more vulnerable.

**Design and Methods:**

We investigated Se status in a cohort of 387 healthy elderly men (median age 77 yrs; inter quartile range 75–80 yrs) in relation to TFTs and BMD. Se status was determined by measuring both plasma selenoprotein P (SePP) and Se.

**Results:**

The overall Se status in our population was low normal with only 0.5% (2/387) of subjects meeting the criteria for Se deficiency. SePP and Se levels were not associated with thyroid stimulating hormone (TSH), free thyroxine (FT4), thyroxine (T4), triiodothyronine (T3) or reverse triiodothyronine (rT3) levels. The T3/T4 and T3/rT3 ratios, reflecting peripheral metabolism of thyroid hormone, were not associated with Se status either. SePP and Se were positively associated with total BMD and femoral trochanter BMD. Se, but not SePP, was positively associated with femoral neck and ward's BMD. Multivariate linear analyses showed that these associations remain statistically significant in a model including TSH, FT4, body mass index, physical performance score, age, smoking, diabetes mellitus and number of medication use.

**Conclusion:**

Our study demonstrates that Se status, within the normal European marginally supplied range, is positively associated with BMD in healthy aging men, independent of thyroid function. Thyroid function tests appear unaffected by Se status in this population.

## Introduction

Selenium (Se) is a nutritional trace element that is essential for the biosynthesis of selenoproteins. Selenoproteins elicit important functions in different processes such as thyroid hormone (TH) homeostasis and antioxidant defence [1, 2]. Patients with severely compromised selenoprotein biosynthesis or massive Se deficiency show a variety of endocrine defects including abnormal thyroid function tests (TFTs) and delayed or impaired bone formation [3–5]. However, it is still a matter of debate if also more subtle changes in Se status are associated with alterations in TFTs and bone mineral density (BMD). This is especially important for elderly who are at risk for malnutrition [6].

Peripheral metabolism of TH levels is predominantly mediated by the selenoenzymes type 1, type 2, and type 3 deiodinase (D1-3), which all contain a selenocysteine in their catalytic centre. An altered metabolism of TH due to low Se status has been proposed as a mechanism for the age-dependent changes in thyroid parameters [1, 7–13].

In 2012 we described a positive association between Se status and BMD in postmenopausal women [9]. No data on the association of Se status and BMD in elderly men are yet available [14–16], despite the knowledge that Se biology and selenoprotein expression show sex-specific differences in rodent models and human studies [17].

For those reasons we investigated the association between Se status, TFTs and BMD in a population of elderly men. Se status was determined by measuring both selenoprotein P (SePP) and plasma Se concentrations. SePP is a liver-derived Se storage and transport protein and is considered the most reliable biomarker of Se status [18].

## Materials and Methods

### Study population

The Zoetermeer study is a cohort study conducted in clinically healthy independently living Caucasian elderly men between 1996 and 2000. The specific design and the effect of thyroid hormone concentrations on disease, physical function and mortality has been reported in 2005 [12]. In brief, individuals were drawn from the municipal register of Zoetermeer, The Netherlands. Inclusion criteria were male sex, age at least 70 years, and a sufficient physical and mental status to visit the study center independently. The Medical Ethics Committee of the Erasmus Medical Center approved the study, which included permission for additional measurements in stored serum and plasma samples. Four hundred and three men participated and gave written informed consent.

The subjects were interviewed by the same person and medical history, smoking status and medication use were recorded. A total of 16 individuals were excluded; 6 individuals on TH replacement, 8 individuals taking the thyroid hormone interfering drug amiodarone, and 2 Se outliers (defined as SePP or Se ≥4 standard deviations from the mean). This resulted in a final population of 387 subjects for analysis.

### Determination of Se status

SePP and Se levels were measured in 2007 in the same plasma samples in parallel and blinded to the characteristics of the participants in a laboratory remote from the study site. A previous stability analysis showed no decline over time [19]. Fluorescence spectroscopy was used for Se determination as described earlier [18]. A commercial human serum standard (Sero AS, Billingstad, Norway) was included for standardization. SePP concentrations were determined by a luminometric immune assay as described [19]. The analyses were conducted in duplicates and inter- and intra-assay variations were <15% during the measurements.

Normal values for SePP and Se were determined in our previous study including 2374 European postmenopausal woman, using the same spectroscopy and immune assay [9].

Reference ranges from another Dutch study in 1987 are not comparable due to the use of another Se assay [20]. There is no association between Se status and sex, but there is a positive association with age [21]. Therefore, all analyses are adjusted for age.

### Thyroid function tests

Blood samples were collected in the morning after an overnight fast. Serum was separated by centrifugation and stored at −40 C. All serum TFTs (thyroid stimulating hormone (TSH), free thyroxine (FT4), thyroxine (T4), triiodothyronine (T3) and reverse triiodothyronine (rT3) were determined using well-established assays as described previously [22].

### Bone Mineral Density

Total BMD was measured using dual-energy x-ray absorptiometry (DEXA) (Lunar, Madison, WI), as were hip BMDs at the femoral neck, trochanter, and Ward’s triangle. Quality assurance for DEXA, including calibration, was performed every morning, using the standards provided by the manufacturer [12].

### Physical performance, body composition and diabetes mellitus

Physical performance was assessed as described by Guralnik et al. [23], including measurements of standing balance, walking speed and ability to rise from a chair. Scores of the tests as well as the summary performance scale were comparable with subjects of the same age group investigated by Guralnik et al. [12, 23].

Height and weight were measured in standing position without shoes. Body mass index (BMI) was calculated as the weight in kilograms divided by the square of the height in meters. The average of two readings was used in the analyses.

Hemoglobin A1c (HbA1c) and fasting glucose levels were determined [24]. Diagnosis of new onset diabetes mellitus (DM) was based on a fasting plasma glucose level ≥7.0 mmol/L and HbA1c ≥6.5 percent [25].

### Statistics

Analyses were performed using SPSS version 23 (SPSS Inc, Chicago, Il). Normal distribution was evaluated using the Kolmogorov-Smirnov test, and variables that were not normally distributed underwent natural logarithmic transformation. Linear regression analyses were used to determine the associations between SePP and Se, TFT’s and BMD. ANOVA analysis was used to present mean values for the lowest and highest Se and SePP quartiles to provide extra insight into the actual effects.

Multivariate linear model was used to correct the association between Se status and BMD for TSH, FT4, BMI, physical performance score, smoking status, known and new onset DM and total number of medication use. All analyses were adjusted for age. A *p*-value <0.05 was considered significant.

## Results

### Baseline characteristics and selenium status

The median age of the population was 77 yrs [inter quartile range (IQR) 75–80 yrs; range 73-94yrs]. The mean SePP concentration was 3.4 mg/L (SD±0.75) and median Se was 92 μg/L [IQR 82–101]. Overall Se status was suboptimal, but only 0.5% (2/387) of subjects met the criteria for Se deficiency (SePP < 2 mg/L and Se < 58 μg/L) [9] (Table 1).

**Table 1:** Baseline characteristics.

	Normal value	N = 387
**Age, yrs, median [IQR]**		77[75–80]
**SePP, mg/L, mean (±SD)**	≥ 2.0	3.44(±0.75)
**Se, μg/L, median [IQR]**	≥ 58	91.9[82.0–101.1]
**TSH, mU/L, median [IQR]**	0.4–4.3	0.95[0.60–1.43]
**FT4, pmol/L, mean (±SD)**	11–25	16.6(±3.1)
**Total BMD, mg/cm2, mean (±SD)**		1169.1(±98.0)
**Smoking, no (%)**		66(17.1%)
**Physical performance score, median [IQR]**	0–12	9[7–10]
**BMI, kg/m**^**2**^**, mean (±SD)**	18.5–25.0	25.4(±3.0)
**DM, no (%)**		44(11.4%)
**Medication, no, median [IQR]**		1[0–2]

**Abbreviations:** BMD, bone mineral density; BMI, body mass index; DM, diabetes mellitus pre-existent and new onset; FT4, free thyroxine; IQR, inter quartile range; medication, total number of medication, N, total number of subjects studied; no, number; yrs, years; SD, standard deviation; Se, Selenium; SePP, selenoprotein P; TSH, thyroid stimulating hormone

Thirty one subjects with known DM and 13 patients with new onset DM were identified. The prevalence of 11.4% is conform the large population based study in the Netherlands including 1,614 patients aged >70 years [26], but lower compared to the age and sex specific prevalence of 16% from the European cohorts combined [27].

### Thyroid function tests

SePP and Se levels were not associated with TFTs (Table 2). TH levels depend not only on the activities of TH-metabolizing enzymes but also, among other things, on thyroid function and plasma TH-binding capacity. Therefore, ratios between plasma TH’s are thought to better reflect tissue deiodinase activities [28]. However, T3/T4, T3/rT3 and rT3/T4 ratios were not associated with Se status either (Table 2).

**Table 2:** Associations between selenium status and thyroid function tests.

TFTs		SePP				Se			
		Linear regression		Quartiles		Linear regression		Quartiles	
	Normal value	*B(SE)*	*P*	Q1 Mean(SE)	Q4 Mean(SE)	*B(SE)*	*P*	Q1 Mean(SE)	Q4 Mean(SE)
**TSH, mU/L**	0.4–4.3	<0.01(0.07)	0.95	1.24(0.10)	1.14(0.10)	<0.01(0.03)	0.98	1.23(0.10)	1.30(0.10)
**FT4, pmol/L**	11–25	0.11(0.21)	0.60	16.4(0.33)	16.7(0.32)	0.01(0.01)	0.11	16.2(0.32)	16.7(0.32)
**T4, nmol/L**	58–128	1.73(1.07)	0.11	79.1(1.68)	81.3(1.64)	0.03(0.05)	0.45	80.1(1.66)	79.2(1.65)
**T3, nmol/L**	1.43–2.51	<0.01(0.02)	0.92	1.45(0.03)	1.41(0.02)	<0.01(0.01)	0.80	1.40(0.02)	1.41(0.02)
**rT3, nmol/L**	0.14–0.34	0.01(0.01)	0.22	0.32(0.01)	0.33(0.01)	<0.01(0.01)	0.96	0.33(0.01)	0.32(0.01)
**T3/T4 x100**	1.42–3.05	-0.05(0.03)	0.11	1.95(0.05)	1.78(0.05)	<0.01(0.01)	0.95	1.82(0.05)	1.89(0.05)
**T3/rT3**	3.12–13.03	-0.15(0.11)	0.30	5.01(0.16)	4.56(0.16)	<0.01(0.01)	0.95	4.69(0.16)	4.72(0.16)
**rT3/T4x100**	0.15–0.44	<0.01(0.01)	0.97	0.41(0.01)	0.41(0.01)	<0.01(0.01)	0.84	0.42(0.01)	0.42(0.01)

The results (*B(SE)* and corresponding *P*-values) of the linear regression analyses of SePP and Se levels versus various TFTs are shown. In addition, mean values for the lowest and highest Se and SePP quartiles are presented to provide extra insight into the actual effects. **Abbreviations:** B, Beta; FT4, free thyroxine; rT3, reverse triiodothyronine; Se, Selenium; (SE), standard error; SePP, selenoprotein P; TFTs, thyroid function tests; TSH, thyroid stimulating hormone; T3, triiodothyronine; T4, thyroxine; Q1, first quartile; Q4, fourth quartile

### Bone mineral density

SePP and Se were positively associated with total BMD and femoral trochanter BMD. Se, but not SePP, was positively associated with femoral neck BMD and Ward's triangle BMD (Table 3). We subsequently constructed a multivariate linear regression model to control for a number of potentially interfering factors including; TSH and FT4, as measures of (mild) thyroid dysfunction, BMI, which is known to be a risk factor for osteoporosis and associated with food intake and smoking which is described to modify the antioxidant effect of Se on BMD [14]. The association of DM, Se status and BMD is still a matter of debate [29]. Therefore subjects with known and new onset DM are included in the model.

**Table 3:** Selenium status and association with bone mineral density.

BMD mg/cm2	SePP						Se					
	Linear regression		Multivariate regression		Quartiles		Linear regression		Multivariate regression		Quartiles	
	*B*(SE) *Unadjusted*	*P Unadjusted*	*B*(SE) *Adjusted*	*P Adjusted*	Q1 Mean(SE)	Q4 Mean(SE)	*B*(SE) *Unadjusted*	*P Unadjusted*	*B*(SE) *Adjusted*	*P Adjusted*	Q1 Mean(SE)	Q4 Mean(SE)
**Total**	15.57(6.68)	0.02*	13.36 (6.09)	0.03*	1147.6(10.1)	1187.6(9.9)	0.87(0.28)	0.001**	0.81(0.26)	0.001**	1155.6(9.9)	1195.6(9.8)
**Femoral neck**	13.42(10.15)	0.14	10.72(9.91)	0.21	855.5(15.3)	899.9(15.1)	1.36(0.42)	0.001**	1.24(0.41)	0.002**	848.6(15.0)	924.5(14.7)
**Femoral trochanter**	26.35(10.23)	0.01*	23.80(9.52)	0.01*	812.7 (15.4)	881.7(15.2)	1.50(0.43)	<0.001***	1.40(0.40)	<0.001***	813.9(15.0)	903.6(14.8)
**Femoral ward**	16.22(11.30)	0.08	12.55(11.14)	0.15	688.6(17.1)	736.4(16.8)	1.38(0.47)	0.003**	1.25(0.47)	0.006**	678.4(16.7)	760.3(16.5)

The results (*B*(SE) and corresponding *p*-values) of the linear regression analyses of SePP and Se levels versus BMD are shown. Multivariate linear model was used to correct for TSH, FT4, age, BMI, physical performance score, smoking status, known and new onset diabetes mellitus and total number of medication use (*B*(SE) adjusted and corresponding adjusted *p*-values). Mean values for the lowest and highest Se and SePP quartiles are presented to provide extra insight into the actual effects. **Abbreviations:**B, Beta; BMD, bone mineral density; BMI, body mass index; FT4, free thyroxine; Se, Selenium; (SE), standard error; SePP, selenoprotein P; TSH, thyroid stimulating hormone; Q1, first quartile; Q4, fourth quartile;

*, *P*<0.05;

**, *P*<0.01;

***, *P*<0.001

To account for the influence of chronic diseases and physical activity we also included the total number of medication use and physical performance score in the model.

After additional adjustment for these factors the positive associations between Se status and BMD remained statistically significant (Table 3).

## Discussion

In the present study we investigated Se status in healthy elderly men in relation to TFT’s and BMD. Although elderly subjects are at increased risk of nutritional deficiencies, with our assay that is well suited to cover low SePP concentration ranges, only a few patients were Se deficient. This is an important finding, especially since Se deficiency is becoming increasingly recognized as a health risk. These results are in agreement with the vast majority of studies that all conclude that European subjects are marginally supplied and on average below the Se concentration needed for full expression of selenoproteins. [30, 31]. This is the first study to show in men that Se status, even within this low normal range, is positively associated with BMD independent of TH status. This is in concordance with our recent findings in elderly postmenopausal women [9].

Although an altered metabolism of TH due to low Se status has been proposed as a mechanism for the age-dependent changes in TFTs [7, 8], extensive profiling of thyroid parameters in the current study did not reveal any association of TFTs with Se status. Our analysis included assessment of T3/T4 and T3/rT3 ratios as a reflection of the peripheral metabolism of TH. We can therefore conclude that our previously reported association between FT4 and rT3 levels with physical performance and/or survival in this cohort is not mediated via Se status [12]. The lack of association between Se status and TFTs in the current study is in line with a randomized controlled trial in elderly in which Se supplements failed to improve thyroid function [10]. In addition, also in Se-deficient transgenic mice, the synthesis and metabolism of TH is surprisingly well maintained [32, 33].

These results are in contrast, however, with our recent study in 1144 healthy euthyroid postmenopausal women in which Se and SePP were inversely associated with FT3 and FT4 and positively associated with the T4/T3 ratio [9]. The Se status of these two populations is comparable. This may point towards a sex-specific difference in this interaction, in line with a number of other sexual dimorphisms in Se metabolism in patients as well as in experimental animals [17]. Notably, expression of D1 strongly differed between male and female rodents via Se-dependent mechanisms affecting protein translation [34]. Unfortunately, no serum rT3 levels were available in the previous study on postmenopausal females, which would have allowed for a better comparison and speculation on the differential effects of Se status and peripheral deiodinase activities between elderly males and females. Discrepancies between the two studies might also be explained by the relatively older age of the current male population (77.8 (±3.6) versus 67.8 (±7.0) yrs). Older subjects may have more co-morbidities which also affect the degradation of TH.

Low Se status is known to be associated with skeletal disease in patients with mutations in selenoproteins (selenocystein insertion sequence binding protein 2), Kashin-Beck osteoarthropathy and women [4, 9]. Also, Se intake appears to be inversely associated with the risk of osteoporotic hip fractures [14]. Women are known to be more vulnerable to osteoporosis [35], but our current findings demonstrate that Se status influences BMD in men as well. Although only two individuals had subnormal Se values, it is very interesting that even in a population with borderline sufficiency there is a significant association with bone mineral density. An effect of TH on BMD could be excluded as correction for thyroid status did not affect the observed associations between Se status and BMD. Some previous clinical studies in healthy women did not demonstrate an association between Se status and BMD, possibly due to a lack of power (77 and 107 subjects) [15, 36]. Mechanistically, SePP has been shown to transport Se to bone, and a receptor-mediated uptake ensures a relatively high bone Se supply even during Se shortage [37]. *In vitro* studies have demonstrated an effect of Se on osteoblast differentiation and subsequent bone resorption by modulating oxidative stress [38, 39].

Our study has a number of potential limitations. Due to the cross-sectional design of the study, causality of the associations found cannot be assessed. We cannot exclude that the voluntary participation of our subjects has resulted in a bias with more health-interested and thus better eating elderly being enrolled. In addition, no data on vitamin D or calcium levels were available. In our previous paper the association of Se status and BMD was not influenced by vitamin D [9]. To the best of our knowledge there is no clear evidence that plasma calcium levels are associated with Se status. PTH, which reflects changes in calcium homeostasis, did not influence the association of Se status and BMD in previous studies either [9]. Therefore, it is not very likely that in the current study in healthy ambulant men, differences in vitamin D or calcium levels have confounded or mediated our results. Finally, while we have shown effects on BMD, no data on fracture risk were available. Future studies should therefore investigate the relation between Se levels and fracture risk, as well as the underlying pathophysiological mechanisms of these observed associations.

## Conclusions

Our study demonstrates that Se status within the low normal range is positively associated with BMD in healthy aging European men, independent of TH function. TFTs appear unaffected by Se status in this population.

## Abbreviations

B: Beta
BMD: bone mineral density
BMI: body mass index
DM: Diabetes Mellitus
FT4: free thyroxine
IQR: inter quartile range
N: number of subjects
*: *p*<0.05
**: *p*<0.01
***: *p*<0.001
rT3: reverse triiodothyronine
SD: standard deviation
Se: selenium
(SE): standard error
SePP: selenoprotein P
TFTs: thyroid function tests
TH: thyroid hormone
TSH: thyroid stimulating hormone
T3: triiodothyronine
T4: thyroxine
